# A Nurr1 ligand C-DIM12 attenuates brain inflammation and improves functional recovery after intracerebral hemorrhage in mice

**DOI:** 10.1038/s41598-022-15178-7

**Published:** 2022-06-30

**Authors:** Keita Kinoshita, Ayaka Yoshimizu, Yusei Ichihara, Keisuke Ushida, Shunsuke Kotani, Yuki Kurauchi, Takahiro Seki, Hiroshi Katsuki

**Affiliations:** 1 Department of Chemico-Pharmacological Sciences, Graduate School of Pharmaceutical Sciences, Kumamoto University, 5-1 Oe-honmachi, Chuo-ku, Kumamoto, 862-0973 Japan; 2 Department of Chemico-Pharmacological Sciences, School of Pharmacy, Kumamoto University, Kumamoto, Japan; 3 Global Center for Natural Resources Sciences, Graduate School of Pharmaceutical Sciences, Kumamoto University, Kumamoto, Japan

**Keywords:** Diseases of the nervous system, Pharmacology, Stroke

## Abstract

We have previously reported that amodiaquine, a compound that binds to the ligand-binding domain of a nuclear receptor Nurr1, attenuates inflammatory responses and neurological deficits after intracerebral hemorrhage (ICH) in mice. 1,1-*Bis*(3′-indolyl)-1-(*p*-chlorophenyl)methane (C-DIM12) is another Nurr1 ligand that recognizes a domain of Nurr1 different from the ligand-binding domain. In the present study, mice were treated daily with C-DIM12 (50 or 100 mg/kg, p.o.) or amodiaquine (40 mg/kg, i.p.), or twice daily with 1400 W (20 mg/kg, i.p.), an inducible nitric oxide synthase (iNOS) inhibitor, from 3 h after ICH induction by microinjection of collagenase into the striatum. C-DIM12 improved the recovery of neurological function and prevented neuron loss in the hematoma, while suppressed activation of microglia/macrophages and expression of inflammatory mediators interleukin-6 and CC chemokine ligand 2. In addition, C-DIM12 as well as amodiaquine preserved axonal structures in the internal capsule and axonal transport function. We also found that C-DIM12 and amodiaquine suppressed the increases of *iNOS* mRNA expression after ICH. Moreover, 1400 W improved neurological function and prevented neuron loss, activation of microglia/macrophages and axonal transport dysfunction. These results suggest that suppression of iNOS induction contributes to several features of the therapeutic effects of Nurr1 ligands.

## Introduction

Intracerebral hemorrhage (ICH) is a severe type of stroke that is triggered by bleeding in the brain parenchyma, followed by development of hematoma toxicity, inflammation, and oxidative stress. Although patients with ICH exhibit contralateral motor and sensorimotor dysfunction, effective drug therapies for ICH are yet unavailable^1^.

Nurr1 (NR4A2), a member of nuclear receptor superfamily, has originally been implicated in the survival and maintenance of midbrain dopaminergic neurons^2^, but this receptor is also expressed in other types of neurons and glial cells in the central nervous system. Indeed, Nurr1 has been shown to play an important role in the regulation of inflammatory gene expression in glial cells, where it acts as a regulator of nuclear factor κB (NF-κB) signals by stabilizing nuclear corepressor proteins^3^. Owing to a unique structure in the ligand-binding domain, Nurr1 has been proposed as a constitutively active receptor that does not require ligand binding for its transcriptional activity^4^. On the other hand, an increasing amount of studies has demonstrated that various endogenous and synthetic compounds can bind to Nurr1 and enhance its transcriptional activity^5,6^. For example, a recent study has revealed that prostaglandins E1 and A1 directly interact with the ligand-binding domain of Nurr1 and stimulate transcriptional function^7^.

Amodiaquine, a 4-aminoquinoline class of antimalarial drug, is a representative case of synthetic compounds that exhibit potent agonistic activity on Nurr1^5^. Notably, amodiaquine was found to protect midbrain dopaminergic neurons in a mouse model of Parkinson’s disease^5^, indicating that synthetic Nurr1 ligands may serve as useful therapeutic options for neurological disorders. Consistent with this idea, several studies examining the effect of amodiaquine on cognitive functions and histological parameters of normal mice^8^ and a mouse model of Alzheimer disease^9^ provided promising results. In this context, we have previously reported that amodiaquine attenuated inflammatory events and behavioral deficits in a mouse model of ICH^10^. Moreover, we recently reported that hydroxychloroquine, which shares a common 4-aminoquinoline structure with amodiaquine, also alleviated behavioral deficits in ICH^11^.

**Figure 1:**
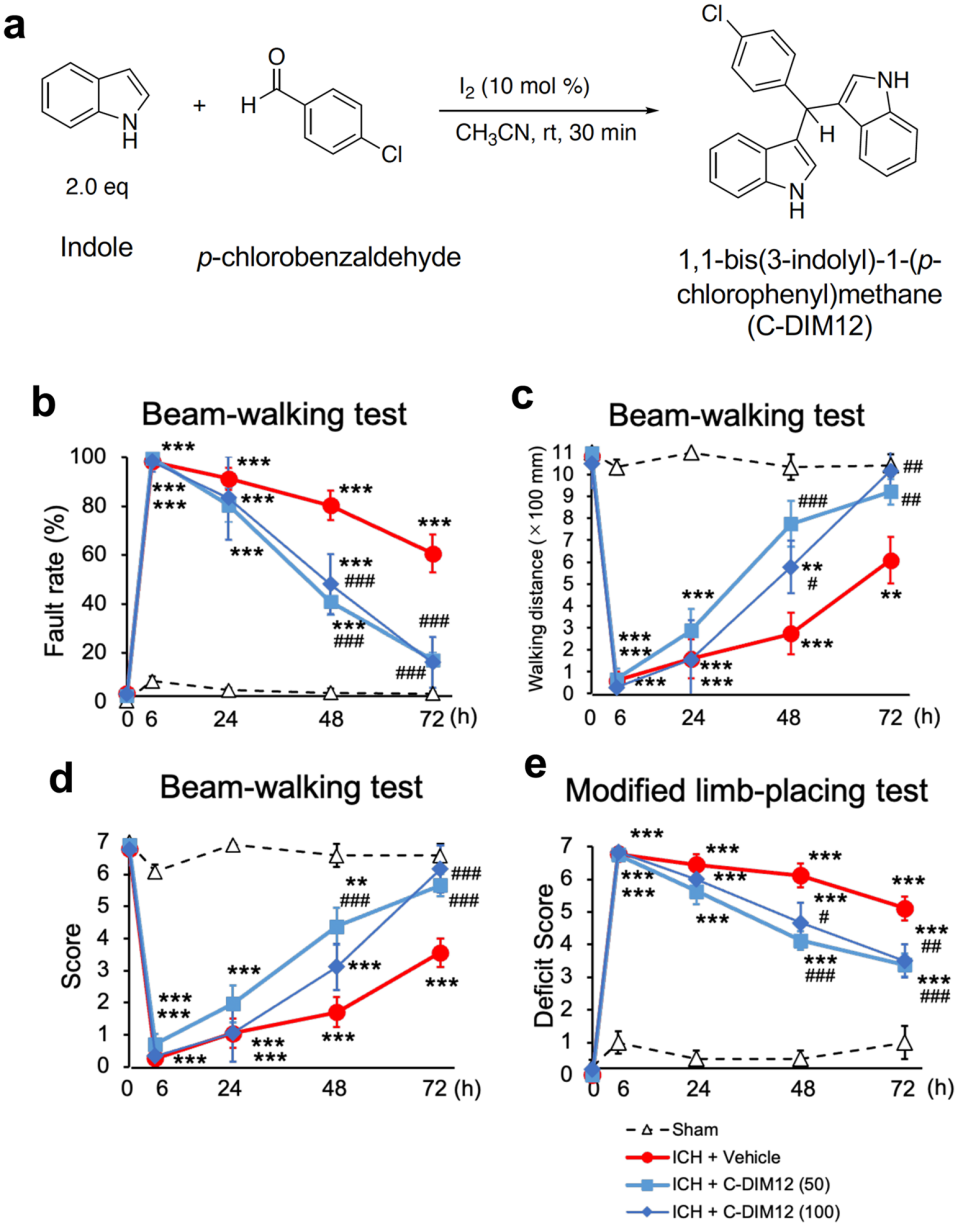
Synthesis of C-DIM12 and effect of C-DIM12 on motor functions of mice after ICH. (**a**) The chemical reaction used for the synthesis of C-DIM12. (**b–d**) Results of performance in the beam-walking test which was evaluated by foot fault rate (**b**), walking distance (**c**) and performance score (**d**). C-DIM12 (50 or 100 mg/kg) or vehicle was orally administered at 3 h, 27 h and 51 h after ICH induction. Concerning the fault rate, significant differences between two groups were observed (two-way repeated measure ANOVA: interactions, *F*_12,92_ = 17.11, *P* < 0.001; time, *F*_4,92_ = 164.4, *P* < 0.001; treatment, *F*_3,23_ = 62.96, *P* < 0.001). Concerning the walking distance, significant differences between two groups were observed (two-way repeated measure ANOVA: interactions, *F*_12,92_ = 9.581, *P* < 0.001; time, *F*_4,92_ = 87.77, *P* < 0.001; treatment, *F*_3,23_ = 23.17, *P* < 0.001). Concerning the performance score, significant differences between two groups were observed (two-way repeated measure ANOVA: interactions, *F*_12,92_ = 11.48, *P* < 0.001; time, *F*_4,92_ = 125.5, *P* < 0.001; treatment, *F*_3,23_ = 32.26, *P* < 0.001). (**e**) Results of performance in the modified limb-placing test. Significant differences between two groups were observed (two-way repeated measure ANOVA: interactions, *F*_12,92_ = 14.92, *P* < 0.001; time, *F*_4,92_ = 180.5, *P* < 0.001; treatment, *F*_3,23_ = 49.86, *P* < 0.001). Number of mice examined in (**b**-**e**) was 4 in sham group, 9 in ICH + vehicle group, 8 in ICH + C-DIM12 (50 mg/kg) group and 6 in ICH + C-DIM12 (100 mg/kg) group, respectively. **P < 0.01, ***P < 0.001 versus sham group, #*P* < 0.05, ##*P* < 0.01, ###*P* < 0.001 versus ICH + vehicle group.

Several derivatives of *para*-phenyl-substituted diindolylmethanes have been reported to constitute another class of Nurr1 ligands and exert anti-inflammatory and neuroprotective effects in a mouse model of Parkinson disease^12^. Especially, 1,1-*bis*(3′-indolyl)-1-(*p*-chlorophenyl)methane (C-DIM12; Fig. 1a) exhibits highly specific activity toward Nurr1^13,14^ and displays favorable pharmacokinetics and anti-inflammatory efficacy with oral dosing, including preferred distribution to the central nervous system as well as neuroprotection against the loss of midbrain dopaminergic neurons^15^. On the other hand, results of a computational analysis indicated that C-DIM12 may bind to the co-activator site, rather than the ligand-binding domain, of Nurr1^15^, while amodiaquine was shown to bind to Nurr1 ligand-binding domain^16^. Moreover, diindolylmethanes such as C-DIM12 and 4-aminoquinolines such as amodiaquine were reported to show different profiles in regulation of Nurr1-dependent gene expression^17^. Therefore, whether or not C-DIM12 produces therapeutic effects on various types of neurological disorders as in the case with amodiaquine is unclear.

In the present study, we examined the effect of C-DIM12 on neurological and pathological parameters of ICH in a mouse model. We also addressed potential role of negative regulation of inducible nitric oxide synthase (iNOS) as the common mechanism of the actions of Nurr1 ligands.

## Results

### C-DIM12 improves neurological outcomes after ICH

Behavioral assessments were conducted before, and 6, 24, 48, and 72 h after induction of ICH. In the beam-walking test, the fault rate increased, whereas the performance score as well as the walking distance decreased substantially after ICH, and then these values gradually recovered during the course of the examination of mice treated with vehicle. Daily oral administration of C-DIM12 (50 mg/kg) for three times from 3 h after induction of ICH markedly alleviated the deficits, and significant differences between vehicle and C-DIM12 groups were detected at 48 and/or 72 h after induction of ICH (Fig. 1b–d). In the modified limb-placing test, the deficit score drastically increased after induction of ICH. C-DIM12 (50 mg/kg) significantly lowered the score at 48 and 72 h after induction of ICH (Fig. 1e). A higher dose of 100 mg/kg C-DIM12 also improved neurological functions significantly, and the degree of improvement was comparable to that observed with 50 mg/kg C-DIM12 (Fig. 1b–e).

### C-DIM12 prevents neural death and activation of microglia/macrophages but not accumulation of activated astrocytes after ICH

**Figure 2:**
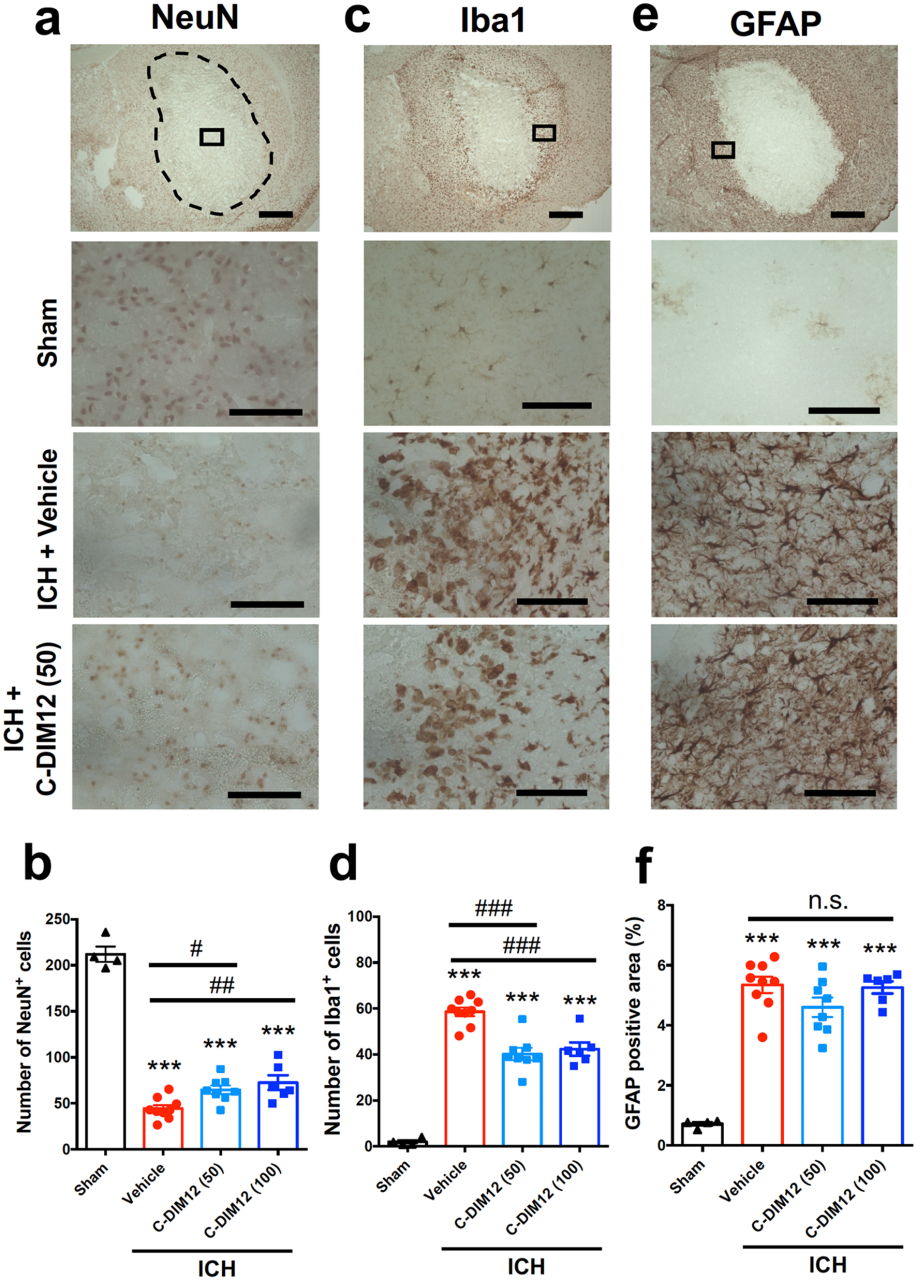
Effect of C-DIM12 on ICH-induced neural death, activation of microglia/macrophages and accumulation of astrocytes. C-DIM12 (50 or 100 mg/kg) or vehicle was orally administered at 3 h, 27 h and 51 h after ICH induction, and immunohistochemical examinations for NeuN, Iba1 and GFAP were conducted on coronal brain sections obtained at 72 h after ICH. (**a**) Representative images of NeuN immunohistochemistry. The top panel shows a low-magnification image of the ipsilateral hemisphere in the brain section obtained from a mouse of ICH + vehicle group. The dashed line indicates the edge of hematoma, and the rectangle indicates the position for cell counting. The other images show magnified views of the regions for quantification obtained from a mouse of sham group, ICH + vehicle group and ICH + C-DIM12 (50 mg/kg) group, respectively. (**b**) Quantitative results of NeuN-positive cells in the central region of hematoma. (**c**) Representative images of Iba1 immunohistochemistry. (**d**) Quantitative results of Iba1-positive cells in the peri-hematoma region. (**e**) Representative images of GFAP immunohistochemistry. (**f**) Quantitative results of GFAP-positive area in peri-hematoma region. Scale bars represent 1 mm in the top images and 100 μm in the other images. Number of mice examined was 4 in sham group, 9 in ICH + vehicle group, 8 in ICH + C-DIM12 (50 mg/kg) group and 6 in ICH + C-DIM12 (100 mg/kg) group, respectively. ***P < 0.001 versus sham group, #*P* < 0.05, ##*P* < 0.01, ###*P* < 0.001, n.s., not significant (ANOVA results: for **b**, *F*_3,23_ = 125.9, *P* < 0.001; for **d**, *F*_3,23_ = 73.72, *P* < 0.001; for **f**, *F*_3,23_ = 41.46, *P* < 0.001).

Next we examined the potential neuroprotective and anti-inflammatory effects of C-DIM12. NeuN immunohistochemistry on coronal sections at 72 h after ICH induction revealed that daily oral administration of 50 mg/kg C-DIM12 significantly prevented neural death in the center of hematoma (Fig. 2a,b). Immunohistochemical detection of Iba1 visualized resident microglia and infiltrating macrophages in the brain, and microglia/macrophages with round or amoeboid shape accumulated in the peri-hematoma area at 72 h after induction of ICH (Fig. 2c,d). Daily oral administration of 50 mg/kg C-DIM12 resulted in a significant decrease in the number of activated microglia/macrophages. On the other hand, 50 mg/kg C-DIM12 did not affect the accumulation of glial fibrillary acidic protein (GFAP)-immunopositive astrocytes in the peri-hematoma region at the same time point (Fig. 2e,f). Similar results were obtained with a higher dose of 100 mg/kg, which prevented neural death, decreased activation of microglia/macrophages but did not affect accumulation of activated astrocytes, and the degree of the effects was not different from that of 50 mg/kg (Fig. 2b,d,f). Based on the results shown in Fig. 1 and Fig. 2, the following series of experiments examined the effects of C-DIM12 at a dose of 50 mg/kg.

### C-DIM12 suppresses ICH-induced mRNA expression of several inflammatory cytokines/chemokines

**Figure 3:**
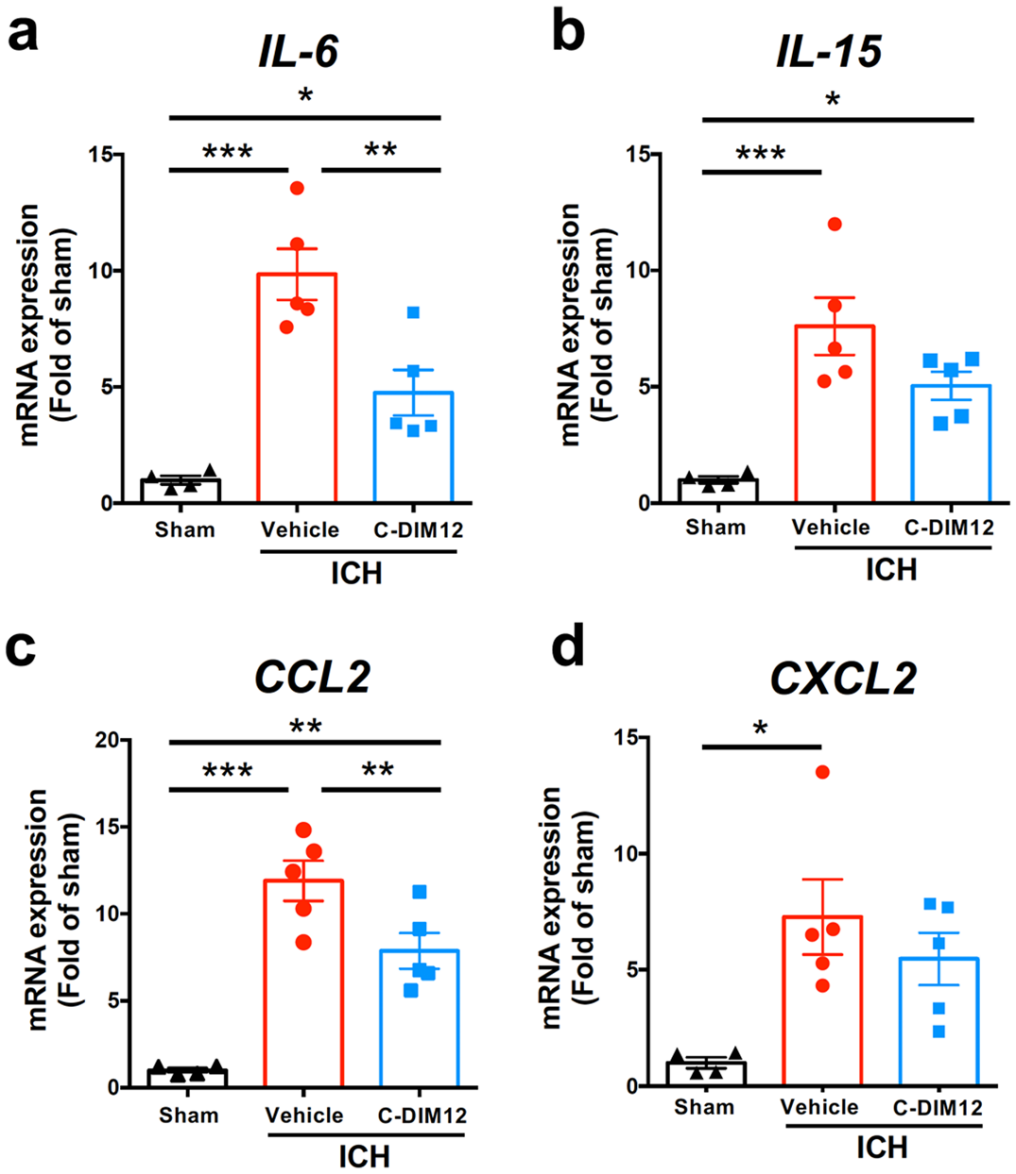
Effect of C-DIM12 on gene expression of pro-inflammatory cytokines and chemokines after ICH. C-DIM12 (50 mg/kg) or vehicle was orally administered at 3 h after ICH induction. Expression levels of IL-6 (**a**), IL-15 (**b**), CCL2 (**c**) and CXCL2 (**d**) mRNAs were quantified at 6 h after ICH. Number of mice examined was 4 in sham group, 5 in ICH + vehicle group, and 5 in ICH + C-DIM12 group, respectively. *P < 0.05, **P < 0.01, ***P < 0.001 (ANOVA results: for **a**, *F*_2,11_ = 22.26, *P* < 0.001; for **b**, *F*_2,11_ = 14.07, *P* < 0.001; for **c**, *F*_2,11_ = 30.44, *P* < 0.001; for **d**, *F*_2,11_ = 6.360, *P* = 0.015).

To verify whether C-DIM12 could affect inflammatory responses associated with ICH, we performed real-time quantitative polymerase chain reaction (RT-qPCR) analysis on the expression of pro-inflammatory cytokines and chemokines. We confirmed robust increases in mRNA expression levels for cytokines *interleukin (IL)-6* and *IL-15* as well as chemokines *CC chemokine ligand 2* (*CCL2)* and *C-X-C motif ligand 2 (CXCL2)*, at 6 h after ICH induction (Fig. 3a–d). Oral administration of C-DIM12 at 3 h after ICH induction significantly suppressed the increase in mRNA expression of *IL-6* and *CCL2* (Fig. 3a,c), but not of *IL-15* and *CXCL2* (Fig. 3b,d).

### C-DIM12 and amodiaquine prevent structural damages and transport dysfunction of axons but not brain edema after ICH

**Figure 4:**
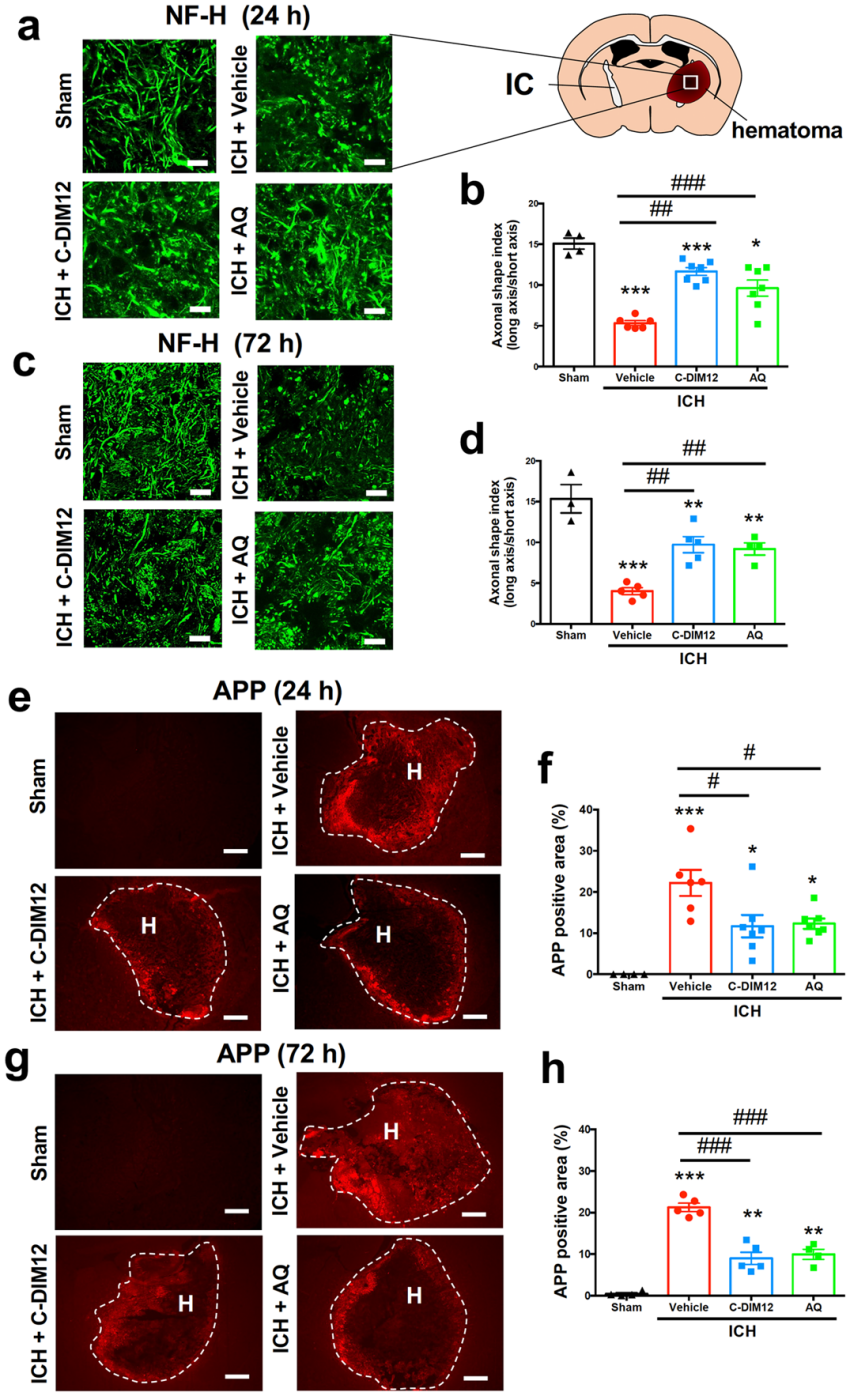
Effects of C-DIM12 and amodiaquine on axonal integrity after ICH. C-DIM12 (50 mg/kg, p.o.), amodiaquine (AQ; 40 mg/kg, i.p.) or vehicle (p.o. or i.p.) was administered at 3, 27 and 51 h after ICH induction. (**a**) NF-H-immunopositive axons in the internal capsule (IC) at 24 h after ICH. Scale bars = 20 μm. (**b**) Quantitative results of axon fragmentation at 24 h. Number of mice was 4 in sham group, 6 in ICH + vehicle group, 7 in ICH + C-DIM12 group and 7 in ICH + AQ group, respectively. (**c**) NF-H-immunopositive axons at 72 h after ICH. Scale bars = 20 μm. (**d**) Quantitative results of axon fragmentation at 72 h. Number of mice was 3 in sham group, 5 in ICH + vehicle group, 5 in ICH + C-DIM12 group and 4 in ICH + AQ group, respectively. (**e**) APP immunohistochemistry at 24 h after ICH. Scale bars = 500 μm. H; hematoma. (**f**) Quantitative results of APP-positive area at 24 h. Number of mice was 4 in sham group, 6 in ICH + vehicle group, 7 in ICH + C-DIM12 group and 7 in ICH + AQ group, respectively. (**g**) APP immunohistochemistry at 72 h after ICH. Scale bars = 500 μm. H; hematoma (**h**) Quantitative results of APP-positive area at 72 h. Number of mice was 4 in sham group, 5 in ICH + vehicle group, 5 in ICH + C-DIM12 group and 4 in ICH + AQ group, respectively. *P < 0.05, **P < 0.01, ***P < 0.001 versus sham group, #*P* < 0.05, ##*P* < 0.01, ###*P* < 0.001 (ANOVA results: for **b**, *F*_3,20_ = 28.79, *P* < 0.001; for **d**, *F*_3,13_ = 22.36, *P* < 0.001; for **f**, *F*_3,20_ = 11.74, *P* < 0.001; for **h**, *F*_3,14_ = 56.61, *P* < 0.001).

Invasion of hematoma into the internal capsule causes axonal dysfunction, which is strongly associated with neurological deficits after ICH^18,19^. Therefore, we next examined the effects of C-DIM12 and amodiaquine on axonal structures in the internal capsule. Neurofilament-H (NF-H) immunoreactivity showed destruction and fragmentation of axonal structures at 24 and 72 h after ICH induction (Fig. 4a,c). Results of quantitative analysis of the axon morphology as the axonal shape index^18^ indicated that deterioration of fibrous structures was significantly attenuated by treatment of either C-DIM12 or amodiaquine (Fig. 4b,d). We also performed amyloid precursor protein (APP) immunohistochemistry because accumulation of APP, a substrate of fast axonal transport, has been conventionally used as a marker of axonal transport dysfunction^18,20^. Immunohistochemical staining indicated that APP accumulated in the region affected by hematoma at 24 and 72 h after ICH induction (Fig. 4e,g). Daily treatment with either C-DIM12 or amodiaquine significantly reduced ICH-induced APP accumulation at both time points (Fig. 4f,h).

We also measured brain water content to examine the effect of drugs on brain edema. The brain tissue ipsilateral to the hemorrhage exhibited higher brain water content than that of the contralateral hemisphere at 72 h after induction of ICH in vehicle-treated mice. Neither C-DIM12 nor amodiaquine showed a significant effect on the brain water content (see Supplementary Fig. S1 online).

### C-DIM12 and amodiaquine suppress ICH-induced mRNA expression of iNOS and NO-related oxidative stress

**Figure 5:**
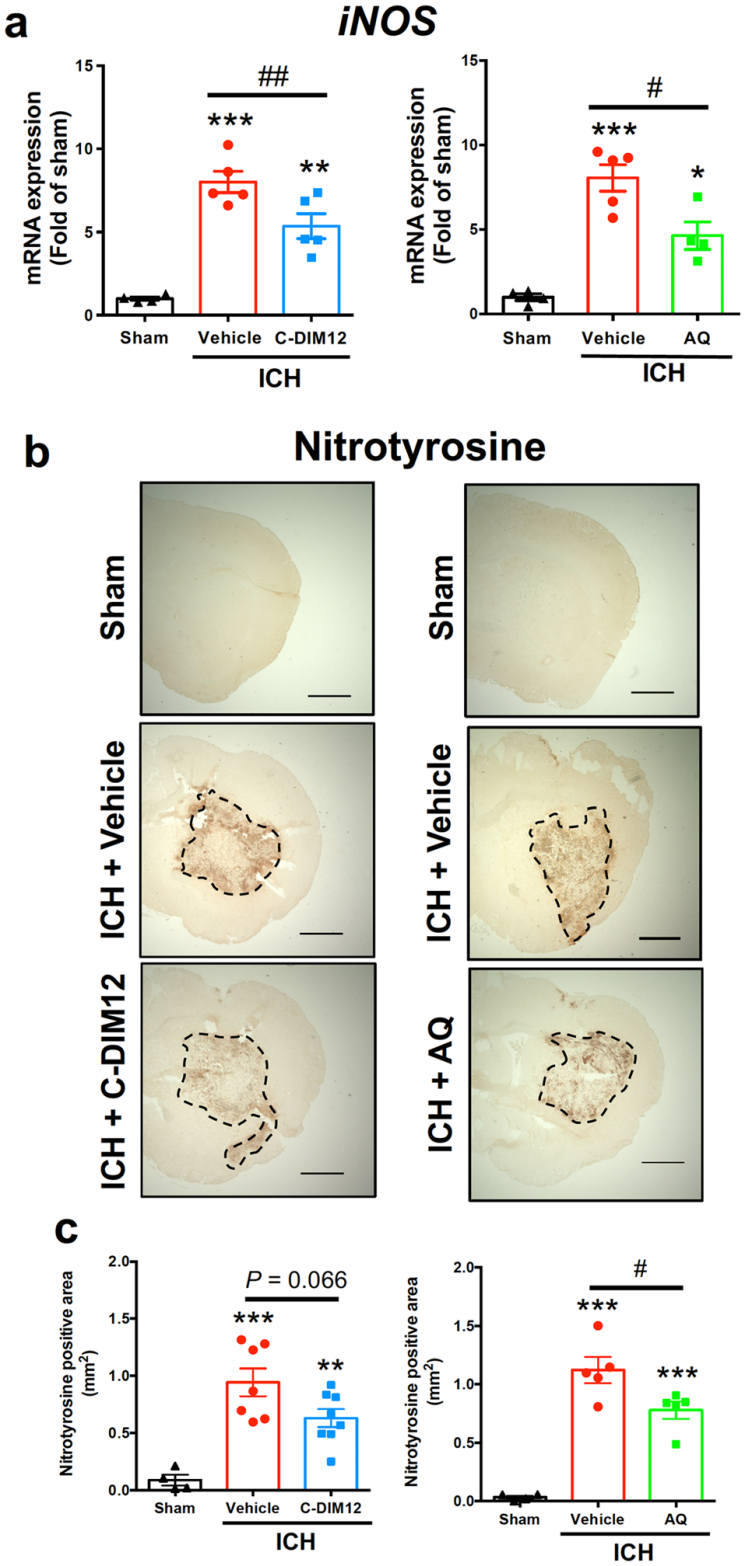
Effects of C-DIM12 and amodiaquine on iNOS expression and NO-related oxidative stress after ICH. C-DIM12 (50 mg/kg, p.o.), amodiaquine (AQ; 40 mg/kg, i.p.) or vehicle (p.o. or i.p.) was administered at 3, 27 and 51 h after ICH induction. (**a**) Expression level of *iNOS* mRNA was quantified at 6 h after ICH. Number of mice examined was 4 in sham group, 5 in ICH + vehicle group, 5 in ICH + C-DIM12 group and 4 in ICH + AQ group, respectively. (**b**) Representative images of nitrotyrosine immunohistochemistry at 72 h after ICH. Scale bars = 1 mm. (**c**) Quantitative results of nitrotyrosine-positive area in the ipsilateral hemisphere. Number of mice examined was 4 in sham group, 7 in ICH + vehicle group (for C-DIM12), 8 in ICH + C-DIM12 group, 5 in ICH + vehicle group (for AQ) and 5 in ICH + AQ group, respectively. *P < 0.05, **P < 0.01, ***P < 0.001 versus sham group, #*P* < 0.05, ##*P* < 0.01 (ANOVA results: for left panel of **a**, *F*_2,11_ = 30.71, *P* < 0.001; for right panel of **a**, *F*_2,10_ = 26.64, *P* < 0.001; for left panel of **c**, *F*_2,16_ = 15.00, *P* < 0.001; for right panel of **c**, *F*_2,11_ = 41.10, *P* < 0.001).

Expression of iNOS, which leads to the production of large quantities of nitric oxide (NO), is implicated in various pathogenic events in the central nervous system^21,22^. Notably, Nurr1 has been reported to suppress transcription of *iNOS* gene and exert an anti-inflammatory effect^3,23^. Accordingly, we examined the effects of C-DIM12 and amodiaquine on the expression of *iNOS*. A significant increase in *iNOS* mRNA expression was observed at 6 h after ICH induction, which was suppressed by treatment with either C-DIM12 or amodiaquine (Fig. 5a). To address the effects of C-DIM12 and amodiaquine on NO-related oxidative stress, we conducted immunohistochemistry against nitrotyrosine^24,25^. The expansion of the area with nitrotyrosine-immunopositive signals observed at 72 h after induction of ICH was considerably suppressed by treatment with C-DIM12 and significantly suppressed by treatment with amodiaquine (Fig. 5b,c).

### iNOS inhibitor decreases NO-related oxidative stress and improves neurological outcomes after ICH

**Figure 6:**
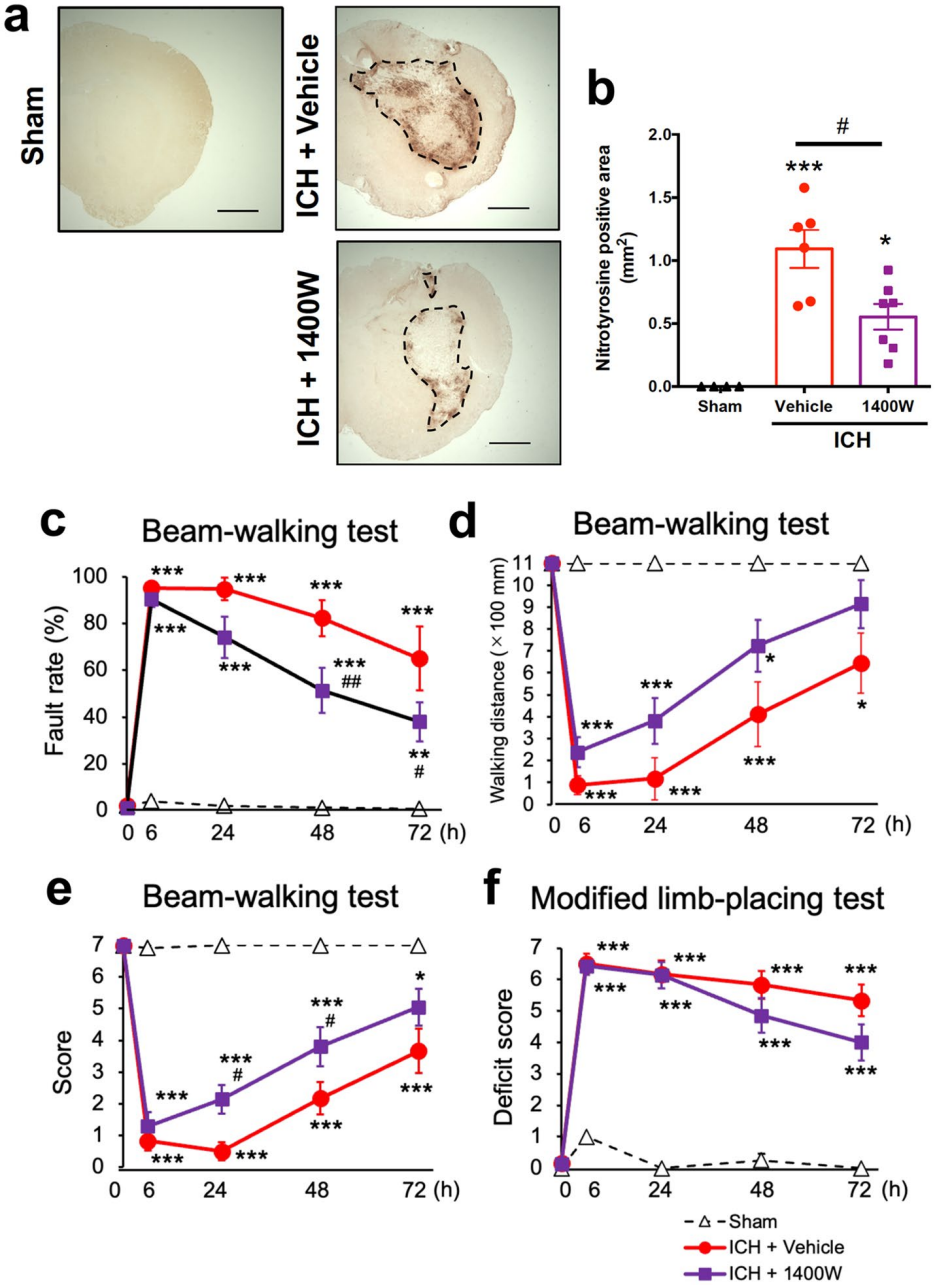
Effect of iNOS inhibitor on NO-related oxidative stress and motor performance after ICH. 1400 W (20 mg/kg) or vehicle was intraperitoneally administered twice daily for six times, from 3 h after ICH induction. (**a**) Representative images of nitrotyrosine immunohistochemistry. Scale bars = 1 mm. (**b**) Quantitative results of nitrotyrosine-positive area in the ipsilateral hemisphere. Number of mice examined was 4 in sham group, 6 in ICH + saline group and 7 in ICH + 1400 W group, respectively. ***P < 0.001 versus sham group, #*P* < 0.05. (ANOVA results: *F*_2,14_ = 15.99, *P* < 0.001) (**c-e**) Results of performance in the beam-walking test evaluated by foot fault rate (**c**), walking distance (**d**) and performance score (**e**). Results of two-way repeated measure ANOVA were as follows. Concerning the fault rate: interactions, *F*_8,56_ = 11.00, *P* < 0.001; time, *F*_4,56_ = 46,80, *P* < 0.001; treatment, *F*_2,14_ = 34.47, *P* < 0.001. Concerning the walking distance: interactions, *F*_8,56_ = 6.830, *P* < 0.001; time, *F*_4,56_ = 28.95, *P* < 0.001; treatment, *F*_2,14_ = 18.18, *P* < 0.001.. Concerning the performance score: interactions, *F*_8,56_ = 11.73, *P* < 0.001; time, *F*_4,56_ = 51.50, *P* < 0.001; treatment, *F*_2,14_ = 35.62, *P* < 0.001. (**f**) Results of performance in the modified limb-placing test. Two-way repeated measure ANOVA: interactions, *F*_8,56_ = 12.66, *P* < 0.001; time, *F*_4,56_ = 69.43, *P* < 0.001; treatment, *F*_2,14_ = 63.77, *P* < 0.001. Number of mice examined in (**c**-**f**) was 4 in sham group, 6 in ICH + vehicle group and 7 in ICH + 1400 W group, respectively. *P < 0.05, **P < 0.01, ***P < 0.001 versus sham group, #*P* < 0.05, ##*P* < 0.01 versus ICH + vehicle group.

In the next series of experiments we examined the effect of a specific iNOS inhibitor 1400W^22,26^ on various pathological parameters associated with ICH, to verify if suppression of iNOS expression contributed to the therapeutic effects of Nurr1 ligands. 1400 W (20 mg/kg) was administered twice daily for six times, the first dose given at 3 h after induction of ICH. As shown in Fig. 6a,b, this dosing regimen effectively suppressed the expansion of the area with nitrotyrosine-immunopositive signals observed at 72 h after induction of ICH. In addition, 1400 W alleviated motor function of mice after ICH. Specifically, a significant ameliorating effect of 1400 W was detected in the fault rate of the beam-walking test at 48 and 72 h after ICH induction (Fig. 6c) and in the performance score at 24 and 48 h after ICH induction (Fig. 6e). The walking distance also tended to be improved by 1400 W treatment, although the difference between groups did not reach statistical significance (Fig. 6d). Deficit score in the modified limb-placing test was slightly and insignificantly decreased by 1400 W treatment (Fig. 6f).

### iNOS inhibitor alleviates histopathological changes associated with ICH

**Figure 7:**
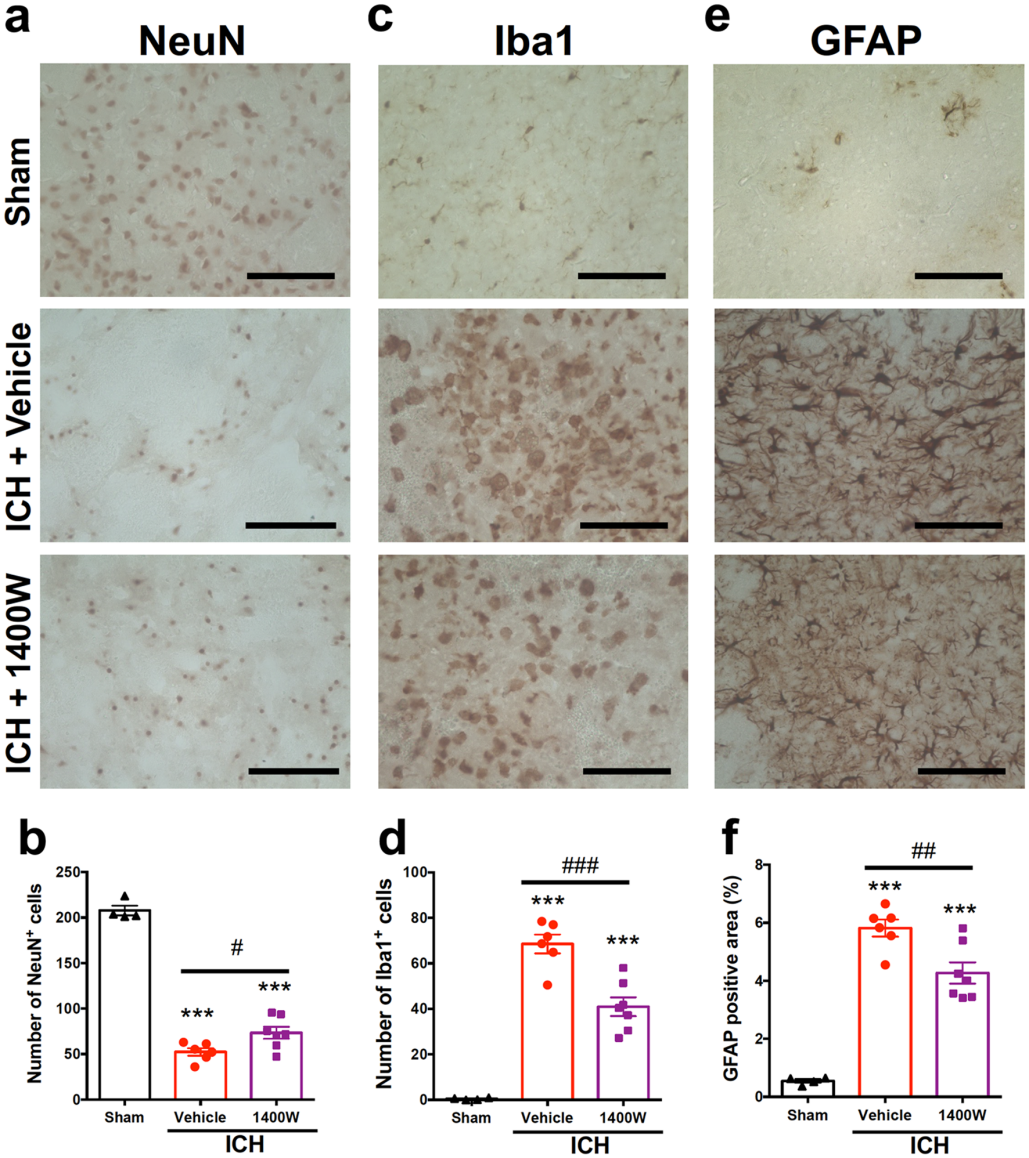
Effect of iNOS inhibitor on ICH-induced neural death, activation of microglia/macrophages and accumulation of astrocytes. 1400 W (20 mg/kg) or vehicle was intraperitoneally administered twice daily for six times, from 3 h after ICH induction. Immunohistochemical examinations for NeuN, Iba1 and GFAP were conducted on coronal brain sections obtained at 72 h after ICH. (**a**) Representative images of NeuN immunohistochemistry. Magnified views of the regions for quantification obtained from a mouse of sham group, ICH + vehicle group and ICH + 1400 W group are shown. Scale bars = 100 μm. (**b**) Quantitative results of NeuN-positive cells in the central region of hematoma. (**c**) Representative images of Iba1 immunohistochemistry. Scale bars = 100 μm. (**d**) Quantitative results of Iba1-positive cells in the peri-hematoma region. (**e**) Representative images of GFAP immunohistochemistry. Scale bars = 100 μm. (**f**) Quantitative results of GFAP-positive area in the peri-hematoma region. Number of mice examined was 4 in sham group, 6 in ICH + vehicle group and 7 in ICH + 1400 W group, respectively. ***P < 0.001 versus sham group, #*P* < 0.05, ##*P* < 0.01, ###*P* < 0.001 (ANOVA results: for **b**, *F*_2,14_ = 172.3, *P* < 0.001; for **d**, *F*_2,14_ = 63.29, *P* < 0.001; for **f**, *F*_2,14_ = 57.42, *P* < 0.001).

We further evaluated the effect of 1400 W by immunohistochemical examinations. 1400 W (20 mg/kg) administered twice daily significantly suppressed the decrease of NeuN-positive cells in the center of hematoma (Fig. 7a,b) and the increase of Iba1-positive activated microglia/macrophages in the peri-hematoma region (Fig. 7c,d) at 72 h after induction of ICH. In addition, 1400 W significantly suppressed the accumulation of GFAP-positive astrocytes in the peri-hematoma region at 72 h after induction of ICH (Fig. 7e,f). On the other hand, 1400 W did not affect the brain water content after ICH induction (see Supplementary Fig. S2 online).

**Figure 8:**
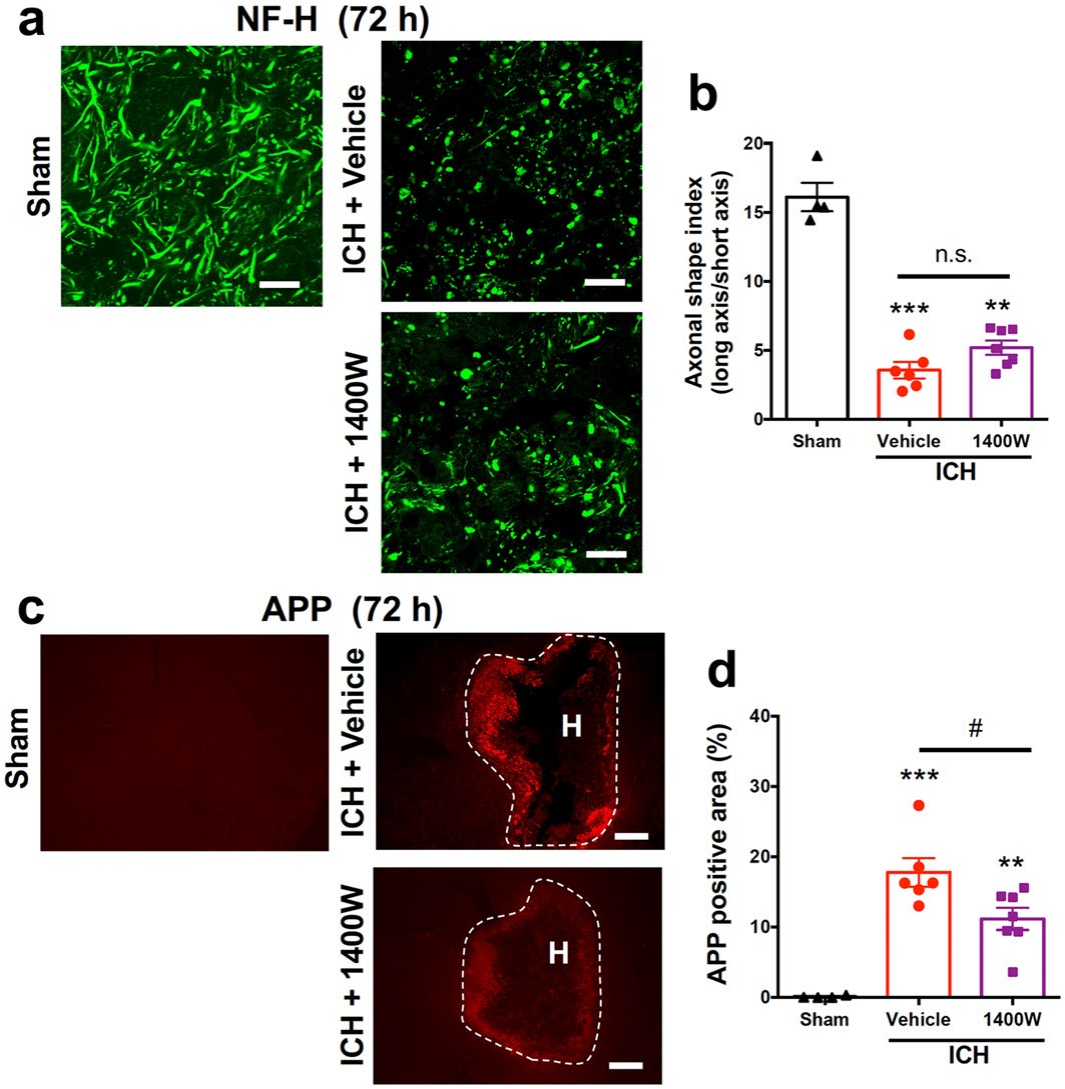
Effect of iNOS inhibitor on axonal integrity after ICH. 1400 W (20 mg/kg) or vehicle was intraperitoneally administered twice daily for six times from 3 h after ICH induction, and immunohistochemical examinations for NF-H and APP were conducted on coronal brain sections obtained at 72 h after ICH. (**a**) Representative images of NF-H-immunopositive axons in the internal capsule within the hematoma. Scale bars = 20 μm. (**b**) Quantitative results of the morphology of axonal fibers as axonal shape index. (**c**) Representative images of APP immunohistochemistry. Scale bars = 500 μm. H; hematoma. (**d**) Quantitative results of APP-positive area. Number of mice examined was 4 in sham group, 6 in ICH + vehicle group and 7 in ICH + 1400 W group, respectively. *P < 0.05, **P < 0.01 versus sham group, #*P* < 0.05, n.s., not significant (ANOVA results: for **b**, *F*_2,14_ = 85.88, *P* < 0.001; for **d**, *F*_2,14_ = 23.16, *P* < 0.001).

Concerning the axons in the internal capsule at 72 h after induction of ICH, administration of 1400 W tended to attenuate the deterioration of fibrous structures detected by NF-H immunoreactivity, but the difference between groups did not reach statistical significance (Fig. 8a,b). On the other hand, 1400 W treatment significantly reduced ICH-induced APP accumulation, indicating that the transport function of axons was ameliorated by iNOS inhibition (Fig. 8c,d).

## Discussion

C-DIM12 has been shown to exert a potent agonistic activity on Nurr1^12–14^. Because this compound shows good bioavailability and crosses the blood–brain barrier, it may become a prototype of therapeutic drugs for various neurological disorders associated with neural damage and neuroinflammation. However, the effect of C-DIM12 has been addressed mainly in experimental models of Parkinson disease^12,15^, except for a few studies on age-associated memory decline^27^ and diabetic retinopathy^28^. Because Nurr1 has been proposed as a promising target for ICH therapy by our previous study demonstrating the effect of another Nurr1 ligand amodiaquine^10^, the present study was aimed to reveal the pharmacological profile of C-DIM12 on a mouse model of ICH.

Recovery of neurological functions impaired by ICH insults is one of the most important parameters for verifying the therapeutic efficacy of drug candidates. We found here that C-DIM12 clearly improved motor functions of mice after ICH, assessed by two different kinds of behavioral tests. The effect of C-DIM12 in these tests was comparable to that of amodiaquine^10^, supporting the idea that Nurr1 serves as a useful therapeutic target for ICH. On the other hand, these Nurr1 ligands did not affect the increase in brain water content after ICH, although brain edema is clinically considered to be an indicator of secondary brain injury after ICH^29^. In this context, we previously reported that several drugs such as retinoic acid receptor agonist^30^ and nicotinic acetylcholine receptor agonist^31^ alleviated motor dysfunction after ICH without suppressing brain edema. The present results add further evidence that the therapeutic effects of drugs on neurological functions after ICH can be produced without accompanying any effect on brain edema.

In our experimental model of ICH, hemorrhage induced by injection of collagenase into the striatum consistently expanded into the adjacent internal capsule. Internal capsule is a white matter region comprised of axon bundles that contain the cortico-spinal tract. Hence, the structural and functional damages of the internal capsule are closely related to the severity of sensorimotor deficits after ICH both in mice^32^ and in humans^33^. Consistent with our previous findings^18^, we confirmed that structural damage of axons in the internal capsule assessed by NF-H immunoreactivity were prominent at 24 h and persistent at least for 72 h after ICH. Importantly, we found that both C-DIM12 and amodiaquine significantly reduced fragmentation of axonal structures after ICH. In addition, we also showed that APP accumulation reflecting dysfunction of fast axonal transport was alleviated by both drugs. The effects of C-DIM12 and amodiaquine on these parameters were evident at both 24 h and 72 h after ICH. These results suggest that preservation of structural and functional integrity of axon tracts by these Nurr1 ligands contributes to the enhanced recovery of motor functions after ICH.

At the same time, we found that C-DIM12 and amodiaquine exhibited some different profiles in their effects on other histopathological parameters. That is, C-DIM12 significantly increased the number of surviving neurons in the striatum affected by hematoma at 72 h after ICH, whereas amodiaquine had minimal effect on neuronal survival in the striatum^10^. Conversely, amodiaquine was effective in reducing the accumulation of activated astrocytes in the peri-hematoma region at 72 h after ICH^10^, but we found no significant effect of C-DIM12 on this parameter in the present study. The precise reasons why C-DIM12 and amodiaquine showed these different profiles of actions are unclear, but two possibilities may be considered. Firstly, these differences may result from different modes of actions of these drugs on Nurr1, where C-DIM12 binds to the co-activator site at the C-terminal^15^ and amodiaquine binds to the ligand-binding domain^5,16^. Consequently, two drugs may stimulate transcription of downstream gene sets different from each other^17^, which should lead to different biological responses of specific cell types. Secondly, off-target actions might be responsible for several effects of these drugs in ICH pathology. For example, C-DIM12 shows a weak activity on another nuclear receptor Nurr77 (NR4A1)^8^, whereas amodiaquine is known to inhibit autophagy^34,35^. In any case, the effects uncommon to C-DIM12 and amodiaquine, such as the protection of striatal neurons in the hematoma and the suppression of astrocyte activation, may not be the major mechanisms of the therapeutic actions of these Nurr1 ligands on ICH. In this context, we showed here that accumulation of activated microglia/macrophages in the peri-hematoma region was significantly inhibited by C-DIM12, and similar effects have also been observed with 4-aminoquinoline Nurr1 ligands amodiaquine^10^ and hydroxychloroquine^11^. These results imply that the therapeutic effects of Nurr1 ligands on ICH are closely associated with their anti-inflammatory effects based on the suppression of activation of microglia/macrophages.

Activated microglia/macrophages are the major sources of pro-inflammatory cytokines and chemokines that may have profound influences on the pathogenic consequences of ICH. We found that ICH-induced upregulation of several pro-inflammatory factors such as IL-6 and CCL2 was significantly attenuated by C-DIM12, confirming the anti-inflammatory effect of the Nurr1 ligand. But we should note here again that C-DIM12 showed different profiles of anti-inflammatory effects than 4-aminoquinoline Nurr1 ligands. For example, upregulation of CXCL2 mRNA was strongly inhibited by amodiaquine^10^, whereas C-DIM12 had no significant effect. On the other hand, hydroxychloroquine did not inhibit induction of cytokines and chemokines including IL-6 and CXCL2, although the drug prevented axonal fragmentation in the internal capsule and alleviated motor deficits after ICH^11^. Hence, the therapeutic effects of diverse kinds of Nurr1 ligands may not be attributable specifically to the suppression of expression of one of these cytokines and chemokines.

Based on these considerations, we examined possible involvement of iNOS as a critical mediator of neuroinflammation. Expression of iNOS is induced under various pathological conditions, and resultant production of excess amount of NO is implicated in the pathogenesis of neurological disorders^36,37^. Notably, C-DIM12 has been shown to inhibit the binding of NF-κB p65 subunit to the *iNOS* promoter in a Nurr1-dependent manner, thereby suppress *iNOS* mRNA expression in mouse microglial BV-2 cell line^23^. In addition, amodiaquine has been shown to inhibit lipopolysaccharide-induced *iNOS* mRNA expression in rat primary microglia^5^. We demonstrated in the present study that C-DIM12 and amodiaquine inhibited ICH-induced upregulation of *iNOS* mRNA. The amount of NO production as reflected by nitrotyrosine immunoreactivity was reduced considerably by C-DIM12 and significantly by amodiaquine, consistent with the idea that suppression of iNOS induction may be a key mechanism of the actions of Nurr1 ligands.

To further address the role of iNOS regulation, we used an iNOS inhibitor 1400 W. We confirmed that 1400 W under the current treatment regimen diminished ICH-associated nitrotyrosine immunoreactivity, to a comparable extent with Nurr1 ligands. In addition, 1400 W inhibited neural death in the hematoma as well as accumulation of activated microglia/macrophages and astrocytes in the peri-hematoma region, indicating that several features of the actions of Nurr1 ligands on these immunohistochemical parameters may be explained by suppressed iNOS expression. Concerning the axon tract integrity, 1400 W significantly prevented dysfunction of axonal transport, although it had no significant effect on fragmentation of axonal structures. In parallel with these effects on axon tracts, 1400 W partially improved the recovery of motor functions after ICH. These results suggest that suppression of iNOS induction is responsible at least in part for the therapeutic effects of Nurr1 ligands. To our knowledge, the present study is the first to demonstrate that pharmacological inhibition of iNOS produces beneficial effects on the symptoms and the pathology of ICH.

In conclusion, C-DIM12 ameliorated neurological functions and preserved axon tract integrity after ICH, and these effects were common with other Nurr1 ligands such as amodiaquine and hydroxychloroquine^10,11^. In addition, we propose that inhibition of iNOS expression underlies some, if not all, of the therapeutic effects of Nurr1 ligands on ICH.

## Methods

### Animals

Male ICR mice (Slc:ICR; Japan SLC, Shizuoka, Japan) at 8 to 10 weeks of age were used in all experiments. They were maintained at constant ambient temperature (22 ± 1 °C) under a 12-h light/dark cycle (light on between 8:00 AM and 8:00 PM) with food and water available ad libitum^38^.

### Induction of intracerebral hemorrhage

The mouse model of ICH was prepared essentially according to the methods described previously^10,38^. After intraperitoneal injection of the combination anesthetic consisting of 0.3 mg/kg medetomidine, 4.0 mg/kg midazolam and 5.0 mg/kg butorphanol, mice were placed in a stereotaxic frame. A 30-gauge needle was inserted into the striatum, with the stereotaxic coordinates of 2.3 mm lateral to the midline, 0.2 mm anterior to the bregma and 3.5 mm deep below the skull. ICH was induced by injection of 0.035 U of collagenase type VII (Sigma-Aldrich, St. Louis, MO, USA) in 0.5 μL of physiological saline at a constant rate of 0.2 μL/min with a microinfusion pump. To prevent backflow of the collagenase solution, the needle was slowly withdrawn from the brain 5 min after the cessation of injection. Body temperature was maintained at 37 °C during the surgery. After surgical operation, mice were returned to their own cage and maintained under the same conditions as pre-operation.

### Drug preparation and administration

For the synthesis of C-DIM12, indole (2.34 g, 20 mmol) was dissolved in acetonitrile (100 mL). To the solution, *p*-chlorobenzaldehyde (1.41 g, 10 mmol) and iodine (126 mg, 1 mmol) were successively added^39^. After stirring for 30 min, the reaction was quenched with 10% aqueous Na_2_S_2_O_3_ solution (50 mL). The mixture was then diluted with ethyl acetate (50 mL), the two-layer mixture was separated, and the organic layer was dried over MgSO_4_. After filtration and evaporation, the obtained crude material was purified by flash column chromatography to give the product (3.52 g, 98% yield) as a red colored amorphous (Fig. 1a).

C-DIM12 was suspended in 0.5% carboxymethyl cellulose (CMC) solution and orally administered to mice at 50 or 100 mg/kg, three times in total. Amodiaquine (cat# CYP526, Cypex Ltd., Dundee, UK) was dissolved in 0.9% physiological saline and administered to mice at 40 mg/kg intraperitoneally, three times in total^12,40^. Administration of these drugs was performed at 3 h after induction of ICH and then daily at a 24-h interval. 1400 W (cat# S8337, Selleck, Houston, TX, USA) was dissolved in 0.9% physiological saline and administered to mice at 20 mg/kg intraperitoneally. The administration of 1400 W was performed at 3 h after induction of ICH and then twice daily at 12-h intervals^26^, six times in total. Control animals received administration of each vehicle. In experiments shown in Fig. 4 and Supplementary Fig. S1 online, three mice received CMC and the other three received saline. There were no obvious differences in histochemical parameters between these mice, and the data were combined as vehicle-treated mice.

### Assessments of motor functions

Motor functions of mice were evaluated by the beam-walking test and the modified limb-placing test before and 6, 24, 48 and 72 h after ICH induction, by investigators blinded to the treatments. In the beam-walking test, mice were trained before surgery to walk on a beam with 15 mm width, 1.1 m length and 50 cm height. The fault rate means the rate of hindlimb foot slips during crossing the beam. The distance score means the number of 10-cm sections across the beam (11 sections in total) that the mouse could reach from an end without falling. The performance score of mice was based on a seven-point scale as previously described^10,30^. In the modified limb-placing test, mouse was suspended 10 cm over a table, and the stretch of forelimbs toward the table was observed and evaluated. Next, the mouse was positioned along the edge of the table with forelimbs suspended over the edge and allowed to move freely. Each limb (forelimb and hindlimb) was pulled down gently, and retrieval and placement were checked. Finally, the mouse was placed toward the table edge to check for lateral placement of forelimb. A total of seven points means the maximal neurological deficits, and 0 point means normal performance^10,30^.

### Immunohistochemical examinations

Immunohistochemistry was performed essentially according to the methods described previously^30^. At 72 h after ICH induction, mice were anesthetized again and perfused transcardially with 30 mL of ice-cold phosphate-buffered saline (PBS) followed by 30 mL of 4% paraformaldehyde. Brains were isolated and preserved in 4% paraformaldehyde overnight at 4 °C, and then soaked in 15% sucrose overnight at 4 °C. After freezing, they were cut into sections of 20 μm thickness obtained every 200 μm, and three sections around the injection site (approximately from + 0.6 mm from bregma to ± 0.0 mm from bregma) were collected for all immunohistochemical examinations except for NF-H and APP. In the case of immunofluorescence histochemistry for NF-H and APP, three sections containing the internal capsule (approximately from − 0.6 mm from bregma to − 1.0 mm from bregma) were collected. Antigen retrieval was achieved by soaking specimens in 100 mM citric acid buffer (pH 8.5) for 30 min at 85 °C. After rinsing with PBS containing 0.3% Triton X-100 (PBS/T), specimens were treated with PBS/T containing blocking serum for 1 h at room temperature, then incubated with primary antibodies overnight at 4 °C. Primary antibodies were rabbit anti-Iba1 antibody (1:500; cat# 019-19,741, FUJIFILM Wako Pure Chemical Co., Osaka, Japan), rabbit monoclonal anti-GFAP antibody (1:500; cat# 12,389, Cell Signaling Technology, Danvers, MA, USA), mouse anti-NeuN monoclonal antibody (1:500; cat# MAB377, Millipore Corporation, Billerica, MA, USA) and rabbit anti-nitrotyrosine (1:500; cat# 06-284, Millipore Corporation). After rinsing with PBS/T, specimens were incubated with the corresponding secondary antibodies for 2 h at room temperature. Biotinylated goat anti-rabbit IgG (1:200 or 1:500; cat# BA-1000, Vector Laboratories, Burlingame, CA, USA) and biotinylated goat anti-mouse IgG (H + L) (1:500; cat# BA-9200, Vector Laboratories) were used as secondary antibodies. After rinsing with PBS/T, specimens were incubated with avidin-biotinylated horseradish peroxidase complex (Vectastain Elite ABC kit; Vector Laboratories) for 1.5 h, and peroxidase was visualized by diaminobenzidine and H_2_O_2_. The number of Iba1-immunopositive microglia/macrophages with activated morphology per 270 × 360 μm^2^ in the peri-hematoma region was counted by an investigator blinded to the conditions of drug treatments^10,38^. The average number of cells from three sections was taken as the value for each mouse. For GFAP immunoreactivity, threshold-based quantification of the immunopositive area was conducted with ImageJ software (National Institutes of Health, Bethesda, MD, USA)^10,41^. The percentage of GFAP-immunopositive area of 270 × 360 μm^2^ in the peri-hematoma region was obtained from each section, and the average percentage from three sections was taken as the value for each mouse. Quantification of NeuN-immunopositive cells in the center of hematoma was performed in a similar manner as that of Iba-immunopositive cells. Nitrotyrosine-positive area was quantified with the use of NIH ImageJ software^30^, and the average percentage from three sections was taken as the value for each mouse. NF-H was immunostained to visualize corticospinal tract in the internal capsule. For NF-H immunostaining, mouse anti-NF-H (1:500; Cell Signaling Technology) and Alexa Fluor 488 donkey anti-mouse IgG (H + L) antibody (1:500; Invitrogen™, Life Technologies Japan, Tokyo, Japan) were used as a primary antibody and a secondary antibody, respectively. To detect accumulation of APP, rabbit anti-beta-Amyloid Precursor Protein (1:100; Invitrogen™) and Alexa Fluor 555 donkey anti-rabbit IgG (H + L) antibody (1:500; Invitrogen™) were used as a primary antibody and a secondary antibody, respectively. Morphological changes in axonal fibers were evaluated from images of NF-H immunostaining. Axonal shape index was calculated as a ratio of length and width of individual immunopositive signals with the use of NIH ImageJ software as previously described^18^. Fifteen fibers in an image of 120 × 120 μm^2^ were randomly selected for the measurement. Threshold-based quantification of APP immunopositive area (%) was conducted with NIH ImageJ software in an image of 270 × 360 μm^2^, and the average percentage from three sections was taken as the value for each mouse.

### RT-qPCR

Quantification of mRNAs by RT-qPCR was performed according to the methods described previously^10,38^. Mice were deeply anesthetized and transcardially perfused with 30 mL ice-cold PBS at 6 h after induction of ICH. A brain slice with 4 mm thickness of the ipsilateral hemisphere that contained the whole hematoma was obtained 2 mm posterior from the frontal pole. The slice was stored in RNAiso Plus reagent (Takara Bio Inc., Shiga, Japan). Reverse transcription of total RNA into cDNA was performed under the conditions of 1 cycle at 37 °C for 15 min and 85 °C for 5 s, using Prime Script™ RT Master Mix (Takara Bio Inc.). Obtained cDNA solution was subjected to real-time PCR (a cycle at 95 °C for 30 s, 40 cycle at 95 °C for 15 s, 55 °C for 45 s, and 72 °C for 30 s) with the use of KAPA SYBR Fast qPCR kit (Japan Genetics Inc., Tokyo, Japan). Glyceraldehyde-3-phosphate dehydrogenase (GAPDH) mRNA was used as internal control. Primer sequences were as follows: IL-6 forward, 5′-TCCAGTTGCTTCTTGGGAC-3′; IL-6 reverse, 5′-GTGTAATTAAGCCTCCGACTTG-3′; IL-15 forward, 5′-TGCGCCCAAAAGACTTGCAGTG-3′; IL-15 reverse, 5′-TCGTCCAACTCTGCAACTGGGC-3′; CCL2 forward, 5′-GAGGAAGGCCAGCCCAGCAC-3′; CCL2 reverse, 5′-TGGGCGTTAACTGCATCTGGC-3′; CXCL2 forward, 5′-CGCTGTCATGCCTGAAGAC-3′; CXCL2 reverse, 5′-CCTTGAGAGTGGCTATGACTTCTG-3′; iNOS forward, 5′-TGCTTTGTGCGGGAGTGTCAGT-3′; iNOS reverse, 5′-CGGAGGATCTCCTGCATTTCT-3′; GAPDH forward, 5′-ACCATCTTCCAGGAGCGAGA-3′; GAPDH reverse, 5′-CAGTCTTCTGGGTGGCAGTG-3′.

### Statistical analysis

All data are presented as mean ± S.E.M. Behavioral data were analyzed by two-way analysis of variance with repeated measures, followed by post hoc comparisons with Bonferroni method (Figs. 1b–e and 6c–f). The other sets of data were analyzed by one-way analysis of variance followed by Tukey’s multiple comparisons test. Statistical analysis was carried out with the GraphPad Prism 6 software (GraphPad, San Diego, CA, USA). Two-tailed probability values < 5% were considered significant.

### Ethics declarations

The present study is reported in accordance with ARRIVE guidelines. All procedures were approved by the Animal Care and Use Committee of Kumamoto University, and animals were treated in accordance with the Guidelines of United State National Institutes of Health (Institute of Laboratory Animal Resources, 1996) regarding the care and use of animals for experimental procedures.

## Supplementary Information

Supplementary Information.

## Data Availability

All raw data in this research are available on reasonable request.
