# Supplementary material for: A Nurr1 ligand C-DIM12 attenuates brain inflammation and improves functional recovery after intracerebral hemorrhage in mice
Source: Sci Rep. 2022 Jun 30;12:11009. doi: 10.1038/s41598-022-15178-7 (PMC9246855; doi:10.1038/s41598-022-15178-7)
Supplement: Supplementary file 1 — Supplementary Information. [file 41598_2022_15178_MOESM1_ESM.pdf]

Supplementary Information

A Nurr1 ligand C-DIM12 attenuates brain inflammation and improves functional recovery after intracerebral hemorrhage in mice

Keita Kinoshita[1], Ayaka Yoshimizu[2], Yusei Ichihara[1], Keisuke Ushida[2], Shunsuke Kotani[3], Yuki Kurauchi[1], Takahiro Seki[1] & Hiroshi Katsuki[1, *]

[1]Department of Chemico-Pharmacological Sciences, Graduate School of Pharmaceutical Sciences, Kumamoto University, Kumamoto, Japan

[2]Department of Chemico-Pharmacological Sciences, School of Pharmacy, Kumamoto University, Kumamoto, Japan

[3]Global Center for Natural Resources Sciences, Graduate School of Pharmaceutical Sciences, Kumamoto University, Kumamoto, Japan

*Corresponding author

Supplementary methods

Measurement of brain water content. Measurement of brain water content was performed mainly according to methods described previously[1]. At 72 h after ICH induction, mice were decapitated under deep anesthesia. Brain was removed from the skull and a coronal brain slice of 4 mm thickness that contained the whole hematoma was obtained 2 mm posterior from the frontal pole (Supplementary Fig. S1a). The brain slice was divided into the ipsilateral side and the contralateral side along the midline. After wet weight of the tissues was obtained, tissues were dried at 75°C for 24 h to give the dry weight. The water content was calculated by the following formula: ((wet weight – dry weight) / wet weight) × 100(%).

Statistical analysis. All data are presented as mean ± S.E.M. Data were analyzed by non-parametrical Kruskal-Wallis test followed by Dunn's multiple comparisons test (Supplementary Fig. S1b) or by one-way analysis of variance followed by Tukey's multiple comparisons test (Supplementary Fig. S2). Statistical analysis was carried out with the GraphPad Prism 6 software (GraphPad, San Diego, CA, USA). Two-tailed probability values < 5% were considered significant.

Reference

1. Matsushita, H. *et al.* A retinoic acid receptor agonist Am80 rescues neurons, attenuates inflammatory reactions, and improves behavioral recovery after intracerebral hemorrhage in mice. *J. Cereb. Blood Flow Metab.* **31**(1), 222–234. https://doi.org/10.1038/jcbfm.2010.80 (2011).

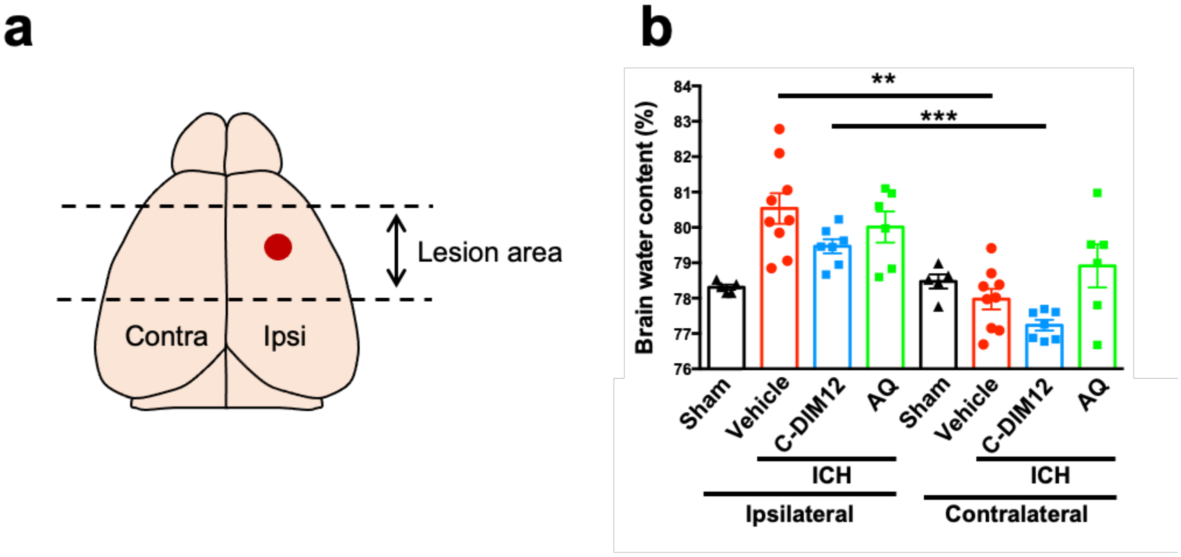

Supplementary Fig. S1. Effect of Nurr1 ligands on ICH-induced brain edema. (**a**) The region used for the measurement of brain water content is shown. (**b**) Results of quantification of brain water content at 72 h after ICH induction are shown. C-DIM12 (50 mg/kg, p.o.), amodiaquine (AQ; 40 mg/kg, i.p.) or vehicle (p.o. or i.p.) was administered at 3, 27 and 51 h after ICH induction. Number of mice examined was 4 in sham group, 9 in ICH +vehicle group, 7 in ICH + C-DIM12 group and 6 in ICH + AQ group, respectively. ** $P < 0.01$, *** $P < 0.001$.

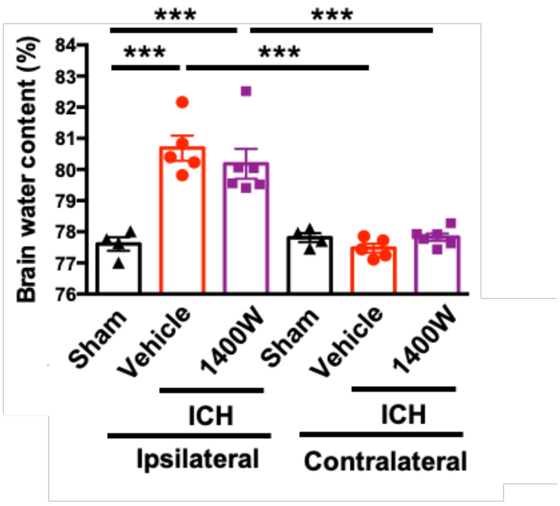

Supplementary Fig. S2. Effect of iNOS inhibitor on ICH-induced brain edema. Results of quantification of brain water content at 72 h after ICH induction are shown. 1400W (20 mg/kg) or vehicle was intraperitoneally administered twice daily for six times, from 3 h after ICH induction. Number of mice examined was 4 in sham group, 5 in ICH +vehicle group and 6 in ICH + 1400W group, respectively. *** $P < 0.001$ (ANOVA results: $F_{5\ 24} = 21.65$, $P < 0.001$).
